# A nomogram based on quantitative MR signal intensity predicts early response to combined systemic treatment in patients with hepatocellular carcinoma

**DOI:** 10.3389/fonc.2025.1527108

**Published:** 2025-03-13

**Authors:** Ran Tao, Haohao Lu, Xiangjun Dong, Qian Qian Ren, Hongjie Fan, Zhaoming Tang, Xiangwen Xia

**Affiliations:** ^1^ Department of Radiology, Union Hospital, Tongji Medical College, Huazhong University of Science and Technology, Wuhan, China; ^2^ Department of Clinical Laboratory, Union Hospital, Tongji Medical College, Huazhong University of Science and Technology, Wuhan, Hubei, China

**Keywords:** hepatocellular carcinoma, nomogram, magnetic resonance imaging (MRI), systemic therapy, signal intensity

## Abstract

**Objective:**

This study aimed to develop and evaluate the value of a nomogram based on quantitative MR signal intensity to predict response to combined systemic therapy of anti-angiogenesis and immune checkpoint inhibitor (ICI) in hepatocellular carcinoma (HCC) patients.

**Methods:**

117 HCC patients who underwent the combined systemic treatment at a tertiary hospital between September 2020 and May 2024 were enrolled and divided into a development cohort (n = 82) and a validation cohort (n = 35). The predictive value of the relative signal intensity attenuation index (rSIAI) based on enhanced MR parameters and laboratory parameters on disease control was evaluated using receiver operating characteristic (ROC) curves, with the determination of optimal cut-off values (COVs) accomplished via Youden’s index. Univariate and multivariable analyses were conducted to evaluate the association between COVs and disease control. The validity of the COVs was further confirmed through chi-square testing and calculation of Cramer’s V coefficient (V). A nomogram was constructed based on the multivariable logistic regression model and evaluated for clinical applicability.

**Results:**

rSIAI from arterial to portal phase (rSI_ap) in combination with peripheral T-cell subset (CD4+) achieved the most accurate predictive performance for outcome compared to rSI_ap or CD4+ alone, with an area under the curve (AUC) of the ROC of 0.845 (95% CI, 0.748-0.915). A nomogram based on rSI_ap and CD4+ was constructed. Calibration and decision curve analyses confirmed the clinical relevance and value of the nomogram.

**Conclusion:**

The nomogram based on rSI_ap has the potential to be a non-invasive tool for predicting disease control in advanced HCC patients who have received combined anti-angiogenesis and ICI therapies.

## Introduction

The amalgamation of anti-angiogenesis therapy and immune checkpoint inhibitors (ICIs) has emerged as a promising regimen for the treatment of advanced hepatocellular carcinoma (HCC) patients (1, 2). Nevertheless, findings from randomized clinical trials have revealed that only a minority of patients exhibit a favorable response to this combined treatment, underscoring the necessity to explore predictive markers capable of discerning individuals likely to benefit from such therapy (3–5). Despite investigations into the predictive value of serological and genetic biomarkers, none have thus far demonstrated clinical utility, often due to their prohibitively high cost or excessive invasiveness, even if offering some degree of assistance.

Magnetic resonance imaging (MRI) is a standard method used for diagnosing, staging, and monitoring treatment in patients with HCC (6–8). Recent studies have shown that the absolute or relative MR signal intensity of lesions can predict outcomes in HCC patients undergoing systemic therapy. For instance, Salvaggio et al. found that a significant decrease in absolute MR signal intensity during the arterial phase was linked to tumor response to sorafenib treatment (9). Additionally, relative MR signal intensity measured during different enhancement phases has been identified as a valuable prognostic factor in prior research (10, 11). However, these studies relied on signal intensity measurements taken at fixed phases, which limits their practical applicability due to the varying time intervals between enhancement phases in different patients. Furthermore, factors influencing tumor response to combined systemic therapy are complex, including characteristics of tumor microvasculature and the proportions of T cell subsets (12, 13). The lack of information regarding peripheral T-cell subsets may also reduce the predictive value of tumor characteristics revealed by imaging features.

In this study, we aimed to evaluate whether the dynamic change in MR signal intensity, which provides insights into the blood supply and venous drainage of lesions, along with peripheral T-cell subsets, can help predict outcomes for patients with HCC who have undergone combined systemic therapy. To our knowledge, no previous study has explored the predictive value of integrating clinical and radiological variables into a nomogram for predicting disease control in advanced HCC patients treated with combined systemic anti-tumor therapy.

## Materials and methods

### Patients

**Figure 1:**
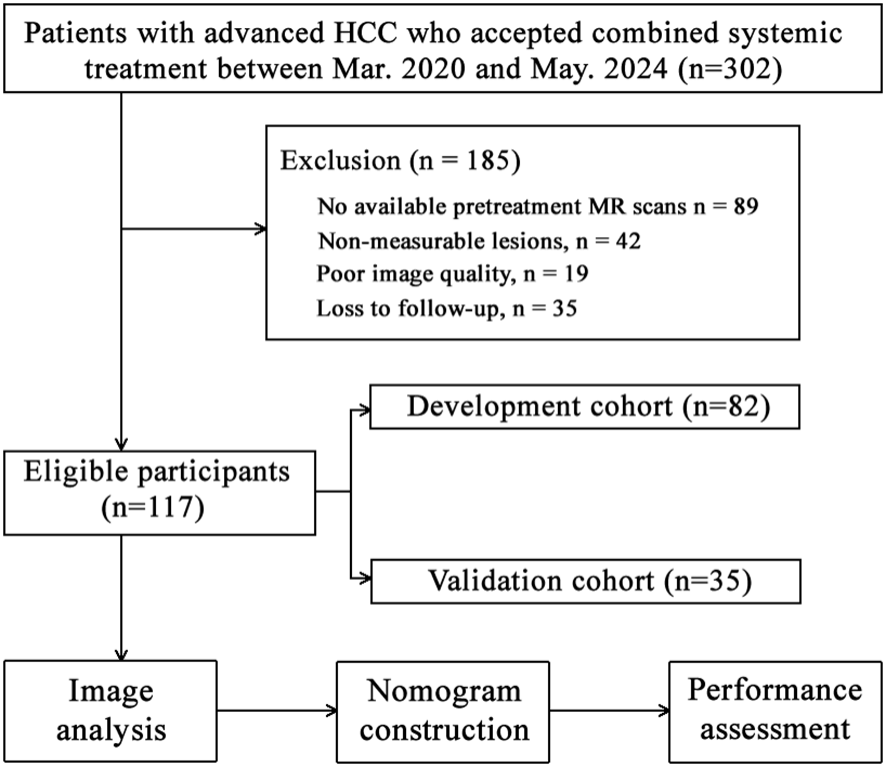
Flowchart of patient selection, model construction, and assessment.

The institutional review board approved the retrospective study conducted at our institute. Informed consent was not required for this study since it involved a retrospective design, and the data used for analysis were anonymized. The study involved patients with HCC staged as Barcelona Clinic Liver Cancer (BCLC) C, as well as those at BCLC B who experienced relapse or progression following local treatment (transarterial chemoembolization or ablation). The diagnosis of HCC was confirmed by pathology or imaging based on the guidelines provided by the European Association for the Study of the Liver European Organization for Research and Treatment of Cancer (EASL-EORT) (14). These patients received a combination of systemic treatment, consisting of anti-angiogenesis agents (Sorafenib, Apatinib, Levatinib, or Donafenib) along with immune checkpoint inhibitors (Camrelizumab, Toripalimab, or Atezolizumab), between September 2020 and May 2024. The exclusion criteria were as follows: (1) previous systemic anti-tumor treatment with anti-angiogenesis or immunotherapy alone or their combination; (2) absence of pretreatment MR scans; (3) incomplete MR data (missing non-enhanced or any of the three enhanced phases); (4) non-measurable lesions or lack of follow-up MR data for immune-modified Response Evaluation Criteria in Solid Tumors (imRECIST) 2 to 4 months after initial treatment; (5) poor image quality. The patient selection flowchart is depicted in **Figure 1**.

Clinical and laboratory data, including age, gender, Eastern Cooperative Oncology Group (ECOG) performance, BCLC stage, Alpha-Fetoprotein (AFP), peripheral cell count (platelet count (PLT), absolute neutrophils count (ANC), absolute lymphocyte count (ALC) and T-cell subpopulation count (CD3+, CD4+, CD8+, and CD4+/CD8+ ratio), and liver function were collected from the electronic database. PLR and NLR are defined as PLT-to-ALC ratio and ANC-to-LNC ratio, respectively.

### Image evaluation

The MR scanners, acquisition parameters, and signal intensity (SI) measurement are listed in the **Supplementary Material**. Two radiologists with 8 years and 10 years of experience in abdominal radiology independently conducted the image analysis. The region of interest (ROI), defined as the enhanced section (the slice showed the longest diameter of the tumor) in arterial phase MR image (represents the primary viable tumor region according to imRECIST), was manually segmented as large as possible along the viable tumor contour and carefully avoiding apparent hemorrhage and necrosis using customizable software (DICOM [Digital Imaging and Communication in Medicine] viewer EV Insite R; PSP Corporation, Tokyo, Japan). The ROIs in the portal and delayed phases were created by copying and pasting the ROI defined in the arterial phase. Signal intensity measurement and tumor response evaluation were performed as previously described with minor modifications (15). Briefly, the software automatically generated the absolute signal intensity of ROIs. Several steps were performed to make the data applicable to other MR brands. Relative SI (rSI) was obtained using the formula: rSI = SI*_tumor_*/SI*_liver_* (SI*_tumor_* and SI*_liver_* represent the absolute SI values of the tumors and the non-tumor liver parenchyma, respectively). rSIAIs, namely rSI_ap (from the arterial to portal-venous phase), rSI_ad (from the arterial to delayed phase), and rSI_pd (from the portal-venous to delayed phase) were calculated for further analysis as follows: rSI_ap = 100 × (rSI*_arterial phase_* – rSI*_portal-venous phase_*)/(Time*_portal-venous phase_* – Time*_arterial phase_*); rSI_ad = 100 × (rSI*_arterial phase_* – rSI*_delayed phase_*)/(Time*_delayed phase_* – Time*_arterial phase_*); rSI_pd = 100 × (rSI*_portal-venous phase_* – rSI*_delayed phase_*)/(Time*_delayed phase_* – Time*_portal-venous phase_*).

**Figure 2:**
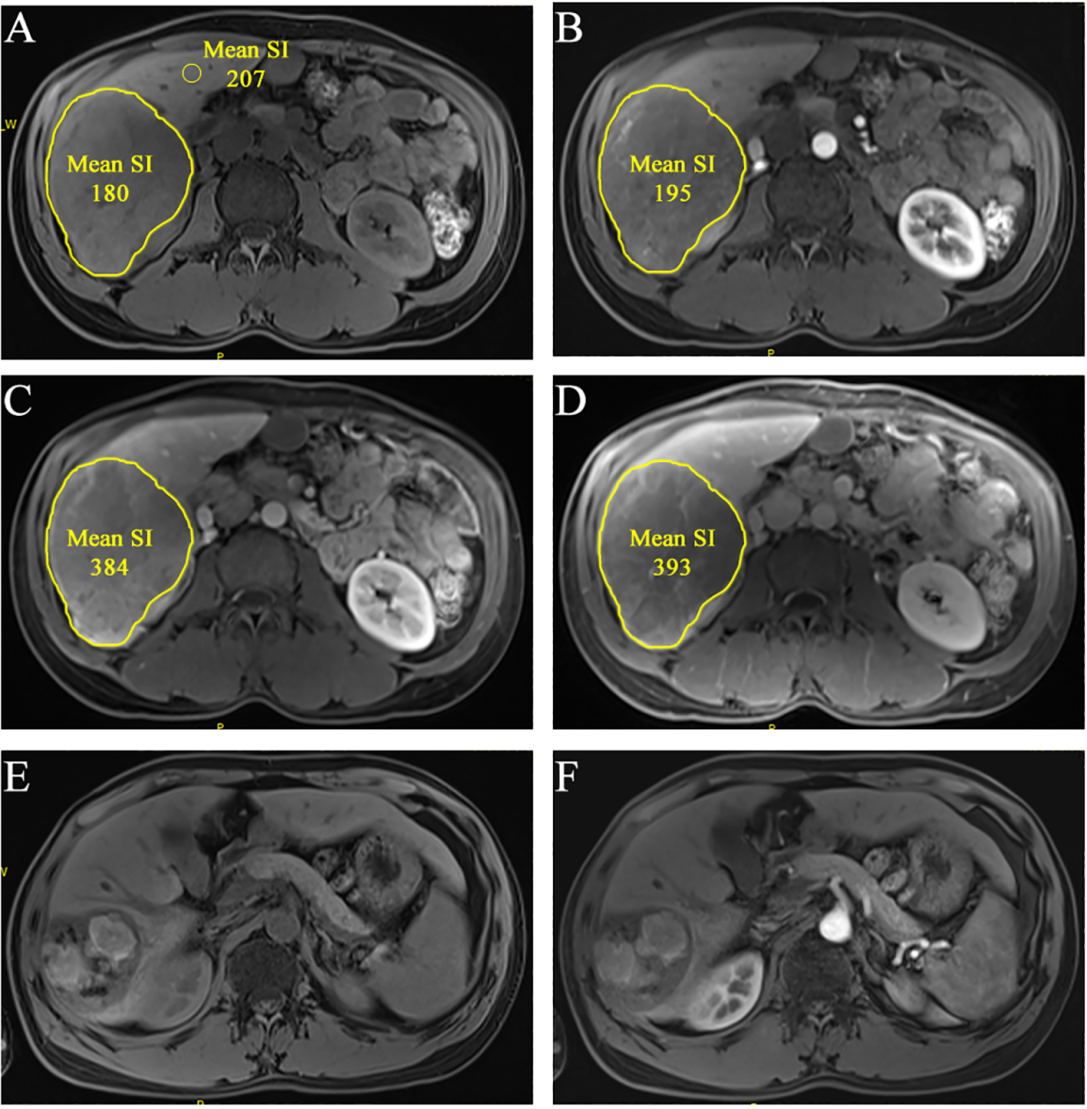
A 36-year-old man of multiple HCCs without macro-vessel invasion or extrahepatic metastasis (staged in BCLC B). The largest lesion is located at S6 liver. ROIs were segmented at the slice that showed the lesion’s longest diameter in pretreatment CEMR image [**(A)**, non-enhanced; **(B)**, arterial phase; **(C)**, portal-venous phase; **(D)**, delayed phase]. The mean SI was automatically generated. rSIAIs were calculated according to the formulas described above. Tumor response was identified as CR according imRECIST because there was almost no enhancement 2.5 months after initial treatment [**(E)**, non-enhanced; **(F)**, arterial phase).

According to immune-modified Response Evaluation Criteria in Solid Tumors (imRECIST) (16), tumor response 2 to 4 months after initial combined treatment is divided into complete response (CR), partial response (PR), progressive disease (PD), and stable disease (SD). Consensus was reached on inconsistencies between readers through consultation (**Figure 2**).

### Statistical analyses

Continuous variables were compared using the Student’s *t*-test or the Mann-Whitney *U* test. Categorical variables were compared using the Pearson *X* ^2^ test or Fisher’s exact test. Inter-observer reliability for measuring signal intensity was assessed using intra-class correlation coefficients (ICCs). The predictive capabilities of quantitative variables on clinical outcomes were compared using receiver operating characteristic (ROC) analysis and the area under the ROC curve. Optimal cut-off values (COVs) were defined as those that exhibited the highest Youden’s index. Multivariate analysis of clinicoradiologic factors for predicting outcomes was performed using binary logistic regression. The independent factors associated with the outcome were further confirmed using the chi-square test and Cramer’s V coefficient (V). A nomogram was constructed based on the results from the final regression analysis. The performance and clinical utility of the nomogram were assessed by the Hosmer-Lemeshow test and decision curve analysis (DCA). Statistical analysis was performed using R software (version 3.4.3; http://www.Rproject.org), SPSS 22.0 statistical package, and MedCalc 11.5 statistics software. A two-sided p-value of less than 0.05 was considered statistically significant.

## Results

### Patient characteristics and tumor depiction

A total of 117 patients were retrospectively enrolled in this study. Overall, the mean age of participants was 56 ± 10, and the percentage of males was 82.1% (96/117). According to the imRECIST criteria, 24 (20.5%) patients (2 CR, 22 PR) were classified as having a tumor response, and 47 (40.2%) patients had stable disease (SD) 2 to 4 months after initial ICI treatment. The remaining 46 (39.3%) patients were identified as having progressive disease (PD). The disease control rate (DCR, CR + PR + SD per total) was calculated as 60.7%. Participants were divided into the development group (n = 82) and the validation group (n = 35) at the ratio of 7:3 in the order of time. The differences in the variables between groups were insignificant (all *p* > 0.05), suggesting a relatively strong homogeneity of the data between the two cohorts (**Table 1**).

**Table 1:** Patient characteristics.

	Development cohort (n = 82)	Validation cohort (n = 35)	*p* value
Sex,male,n (%)	68 (82.9%)	28 (80.0%)	0.794
Age	56 ± 11	55 ± 11	0.827
ECOG			1.000
0	24 (29.3%)	10 (28.6%)	
1	58 (70.7%)	25 (71.4%)	
BCLC stage			1.000
BCLC B	29 (35.4%)	12 (34.3%)	
BCLC C	53 (64.6%)	23 (65.7%)	
Tmax (mm)	59.6 (7.9-196.3)	54.3 (6.6-153.6)	
AFP			0.410
>400	33 (40.2%)	11 (31.4%)	
=<400	49 (59.8%)	24 (68.6%)	
Liver function			
ALT	41 (12-100)	47 (19-118)	0.562
Tbil	15.9 (5.8-55.2)	13.6 (7.9-56.7)	0.493
ALB	35.8 ± 4.6	32.4 ± 4.9	0.671
Blood cell count			
PLT	180 (64-409)	159 (72-313)	0.117
ANC	3.7 (1.4-8.2)	3.5 (1.6-8.1)	0.862
ALC	1.3 (0.4-2.4)	1.3 (0.4-2.5)	1.000
PLR	135.2 (33.8-681.7)	129.5 (36.9-624.1)	0.826
NLR	2.5 (1.0-13.7)	2.6 (1.0-15.2)	1.000
Lymphocyte count			
CD3+	67.6 ± 11.5	69.9 ± 12.1	0.712
CD4+	39.6 ± 11.0	37.4 ± 11.0	0.658
CD8+	20.9 (7.5-58.2)	22.4 (7.5-65.2)	0.613
CD4+/CD8+ ratio	1.84 (0.42-5.36)	1.81 (0.52-4.27)	0.663
Tumor response			0.703
CR	2 (2.4%)	0 (0)	
PR	14 (17.1%)	8 (22.9%)	
SD	34 (41.5%)	13 (37.1%)	
PD	32 (39.0%)	14 (40%)	
Objective response rate	19.5%	22.9%	0.803
Disease control rate	61.0%	60.0%	1.000

### Obtaining standard COVs of parameters for predicting disease control

The inter-observer agreement between the two radiologists revealed excellent consistency in determining signal intensity values, with intra-class correlation coefficient (ICC) values ranging from 0.9429 to 0.998 (**Supplementary Table S1**). The details of the rSIAIs (rSI_ap, rSI_ad, and rSI_pd) are shown in **Supplementary Figure S1**.

**Figure 3:**
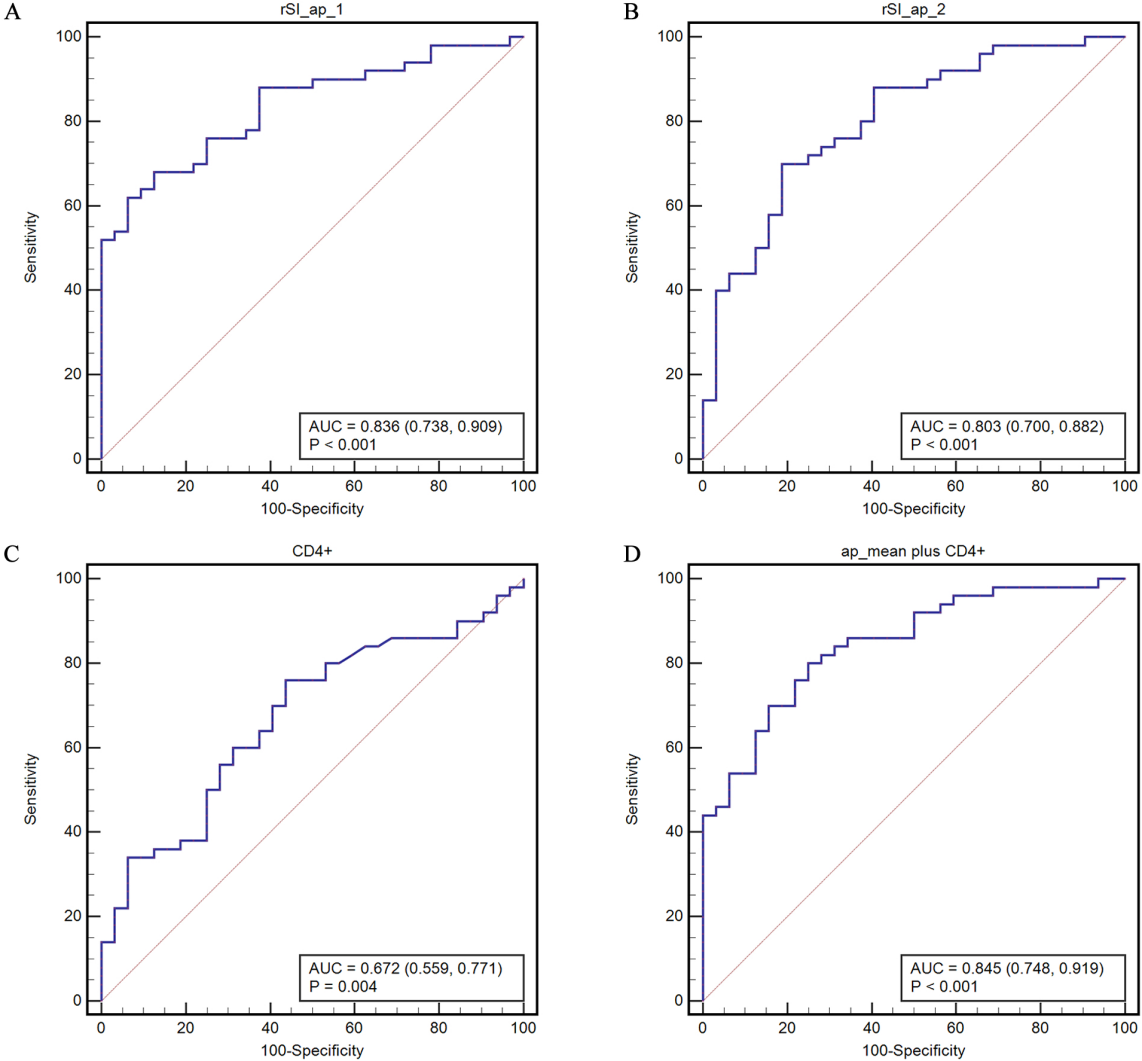
ROC curves for predicting disease control based on the COVs of rHU-ap **(A, B)** of the two observers, CD4+ **(C)**, and their combination **(D)**. rSI_ap_mean represents the mean value of rSI_ap determined by the two observers.

Analysis of the area under the ROC curve (AUC) revealed that the predictive ability of the rSI_ap for disease control is significantly higher than that of rSI_ad (p values were 0.0048 for observer 1 and 0.019 for observer 2). The AUCs were 0.836 (95% CI, 0.738 - 0.909) and 0.803 (95% CI, 0.700 - 0.882) for rSI_ap and 0.706 (95% CI, 0.595 - 0.801) and 0.655 (95% CI, 0.542 - 0.757) for rSI_ad by two observers, respectively. The AUC of CD4+ alone is 0.672 (95% CI, 0.559 - 0.771). When combined with rSIAIs, the AUCs were 0.845 (95% CI, 0.748 - 0.915) (rSI_ap mean plus CD4+) and 0.727 (95% CI, 0.618 - 0.820) (rSI_ad mean plus CD4+), respectively (**Figure 3**, **Supplementary Figure S2**).

The highest Youden’s index was then used to determine the optimal cut-off values (COVs) of the AUCs. The COVs are 0.9819 and 1.3414 for rSI_ap and 0.3188 and 0.278 for rSI_ad, as determined by the observers, respectively. The COV of CD4+ is 35.19. The corresponding sensitivity, specificity, and positive and negative predictive values (PPV, NPV) for the COVs are listed in **Table 2**.

**Table 2:** The corresponding sensitivity, specificity, positive and negative predictive values (PPV, NPV) for the parameters.

	COV of rSI_ap		COV of rSI_ad		COV of CD4+
	Observer 1 (AUC=0.836, COV=0.9819)	Observer 2 (AUC=0.803, COV=1.3414)	Observer 1 (AUC=0.706, COV=0.3188)	Observer 2 (AUC=0.655, COV=0.278)	(AUC=0.672, COV=35.19)
Sensitivity	62.00%	70.00%	68.00%	42.00%	76%
	47.2%-75.3%	55.4%-82.1%	53.3%-80.5%	28.2%-56.8%	61.8%-86.9%
Specificity	93.75%	81.25%	71.87%	87.50%	56.25%
	79.2%-99.2%	63.6%-92.8%	53.3%-86.3%	71.0%-96.5%	37.7%-73.6%
PPV	93.95%	85.38%	79.08%	84.01%	73.10%
NPV	61.20%	63.39%	58.95%	49.10%	59.98%

### Association between parameters and disease control

Univariate analyses revealed statistically significant differences in the COVs for rSI_ap and rSI_ad observed by independent observers, as well as the CD4+ count, between patients with disease control (control group) and those with disease progression (non-control group) (**Table 3**). Multivariable binary logistic analysis was performed using the variables with a *p*-value less than 0.2 in univariate analyses. The results showed that the COVs for rSI_ap and CD4+ were statistically associated with disease control (rSI_ap, Odds ratio: 4.692 and 4.736, 95% CI: 2.172 - 10.352 and 1.6 - 14.02, *p*-value < 0.001 and 0.005; CD4+, Odds ratio: 0.338, 95% CI: 0.122 - 0.935, *p*-value of 0.037). However, the correlation between rSI_ad and disease control was not statistically significant (p = 0.057 and 0.097).

**Table 3:** Univariate and multivariate analyses of predictors of disease control.

Variable	DC(n=50)	Non_DC (n=32)	*p* (Univariate)	*p* (Multivariate)	Odds Ratio	90% CI for Odds Ratio
Sex,male,n (%)	41 (82%)	27 (84.4%)	1.000			
Age	57 ± 11	54 ± 11	0.241			
ECOG			0.621			
0	16 (32%)	8 (25%)				
1	34 (68%)	24 (75%)				
BCLC stage			0.157			
BCLC B	21 (42%)	8 (25%)				
BCLC C	29 (58%)	24 (75%)				
AFP			0.490			
>400	22 (44%)	11 (34.4%)				
=<400	28 (56%)	21 (65.6%)				
Liver function						
ALT	45 (12-100)	44 (23-89)	0.614			
Tbil	17.7 (5.8-55.2)	17.1 (6.7-42.3)	0.578			
ALB	35.8 ± 4.7	35.8 ± 4.5	0.978			
Blood cell count						
PLT	194 (64-397)	198 (66-409)	0.827			
ANC	3.7 (1.5-7.0)	4.2 (1.4-8.2)	0.256			
ALC	1.4 (0.4-2.4)	1.3 (0.4-2.4)	0.354			
PLR	161.3 (33.8-488.9)	187.4 (60.7-681.7)	0.503			
NLR	3.3 (1.0-9.1)	4.1 (1.1-13.7)	0.272			
Lymphocyte count						
CD3+	69.3 ± 11.0	64.8 ± 11.9	0.080			
CD4+	42.1 ± 11.5	35.8 ± 9.2	0.011	0.037	0.338	0.122-0.935
CD8+	21.6 (9.0-58.2)	23.9 (7.5-41.9)	0.138			
CD4+/CD8+ ratio	1.91 (0.42-5.36)	1.75 (0.73-3.77)	0.089			
B cell	10.0 (1.6-29.5)	8.8 (0.4-30.6)	0.332			
rSI_ap						
OB1	1.02 (-0.06-3.90)	2.20 (0.93-9.59)	<0.001	<0.001	4.692	2.127-10.352
OB2	1.05 (-0.12-2.93)	2.22 (0.28-11.29)	<0.001	0.005	4.736	1.6-14.02
rSI_ad						
OB1	0.35 (-0.06-2.26)	0.55 (0.09-2.12)	0.002	0.057	3.534	0.965-12.939
OB2	0.43 (-0.24-2.51)	0.63 (0.05-2.29)	0.018	0.097	2.307	0.859-6.196
rSI_pd						
OB1	0.12 (-0.30-3.06)	0.08 (-0.35-0.91)	0.834			
OB2	0.22 (-0.89-3.27)	0.24 (-0.31-1.66)	0.808			

### Further confirmation of the COVs for rSI_ap and CD4+ for predicting disease control

The COVs for rSI_ap, as defined by independent observers along with their mean value, exhibited a moderate to high association with disease control both in the development cohort (V = 0.33 - 0.555, *p* < 0.01) and in the validation cohort (V = 0.322 – 0.525, *p* < 0.05). Similarly, the COVs for CD4+ demonstrated a moderate association with disease control in the two cohorts (V = 0.322 and 0.319, *p* < 0.05) (**Table 4**).

**Table 4:** Predictors defined from independent observers were grouped through different standards.

Readers	Development cohort			Validation cohort		
	*X*2	Cramer’s V coefficient	*p* value	*X*2	Cramer’s V coefficient	*p* value
rSI_ap:0.9819						
OB 1	25.22	0.555	<0.001	23.26	0.518	<0.001
OB 2	8.94	0.330	0.003	8.56	0.322	0.017
rSI_ap:1.3414						
OB 1	17.33	0.460	<0.001	18.55	0.473	<0.001
OB 2	20.50	0.500	<0.001	22.47	0.525	<0.001
rSI_ap:1.1617						
OB 1	18.09	0.470	<0.001	17.42	0.457	<0.001
OB 2	12.29	0.387	<0.001	11.64	0.384	<0.001
CD4+:35.19	8.747	0.327	0.003	8.472	0.319	0.021

### Nomogram construction, validation, and clinical utility assessment

**Figure 4:**
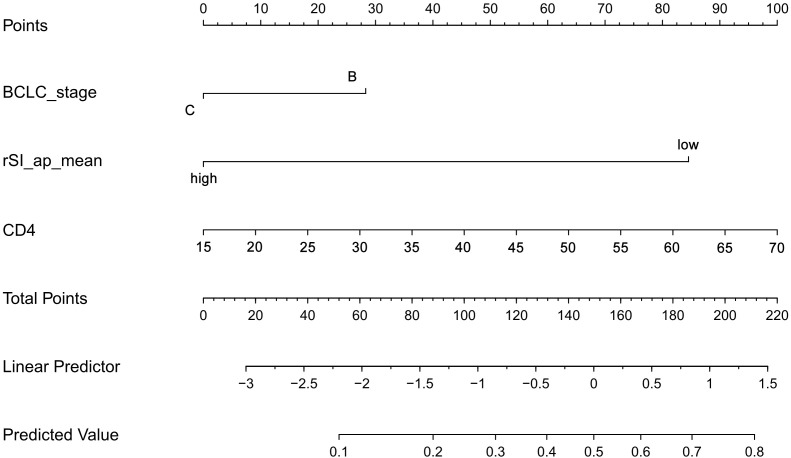
Nomogram for predicting disease control rate (DCR) based on CD4+, rSI_ap, and BCLC stage.

We constructed a nomogram, which incorporated the two significant risk factors (CD4+ and mean value of rSI_ap determined by the two observers) based on the final regression analysis and a factor (BCLC_stage) with clinical significance to predict disease control to systemic therapy. The probability of DCR could be estimated by projecting the total score on the lower total point scale (**Figure 4**).

**Figure 5:**
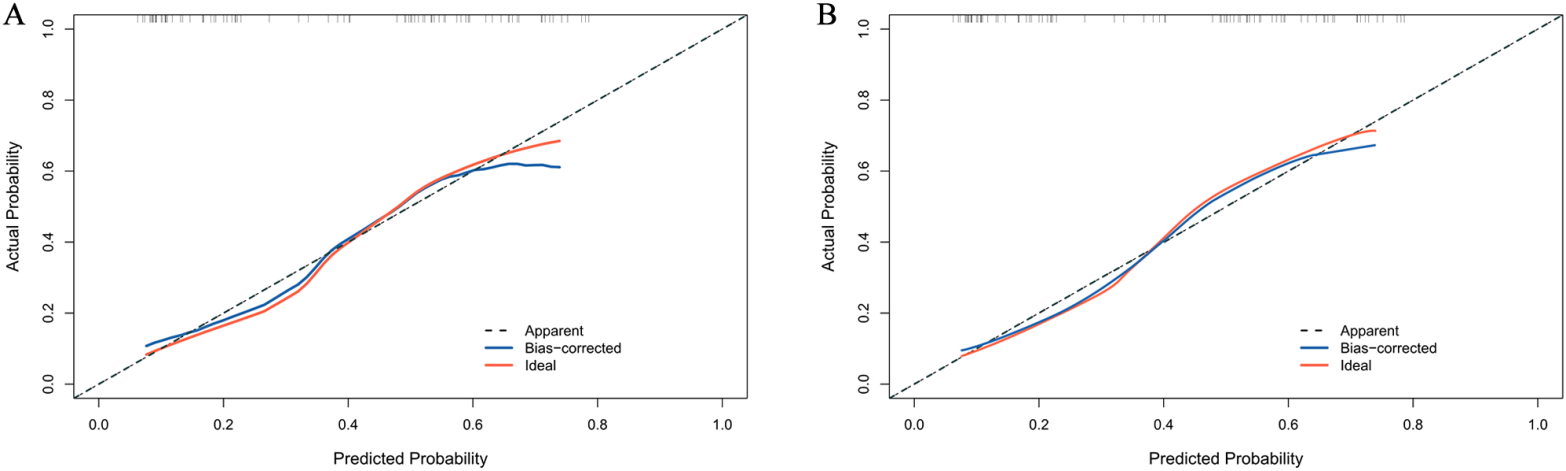
Calibration curve for DCR probability in the development cohort **(A)** and validation cohort **(B)**.

The calibration curve of the nomogram for the probability of DCR demonstrated good agreement between prediction and observation of the combined model both in development and validation cohorts (**Figures 5A, B**). The nomogram model fit well with the data because the Hosmer-Lemeshow test with a calibration curve showed a p-value of more than 0.05 in both cohorts (p = 0.637 and 0.426).

**Figure 6:**
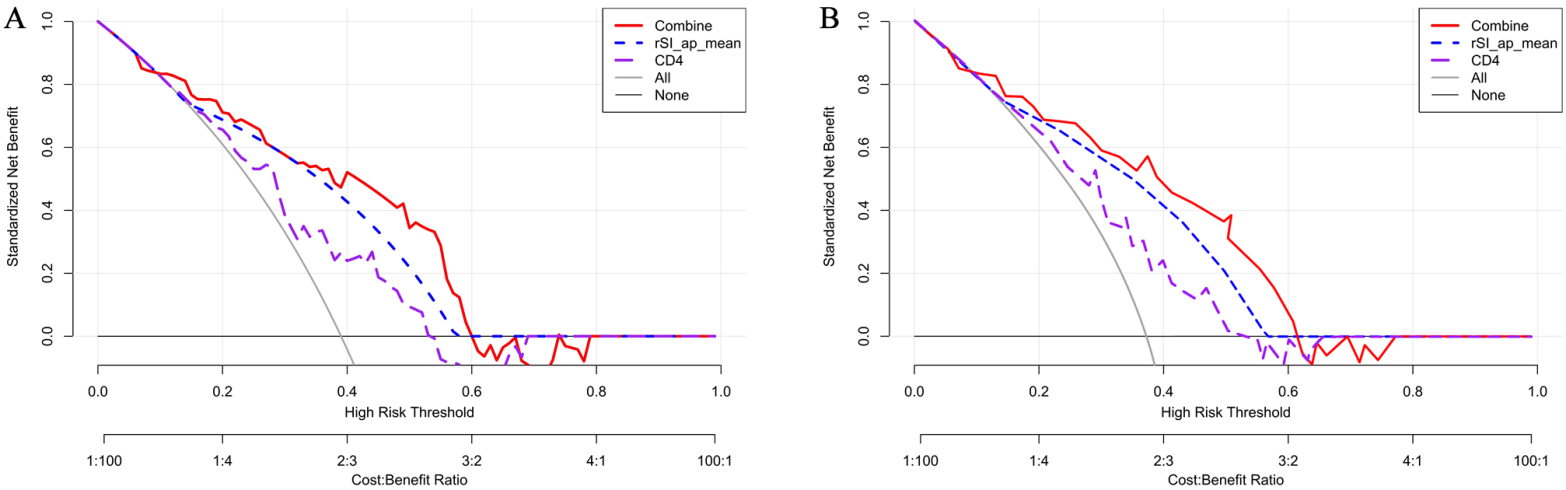
Decision curve analysis for the development cohort **(A)** and validation cohort **(B)**.

The clinical utility of the nomogram was further assessed using decision curve analysis (DCA). As presented in **Figures 6A, B**, using the combined model to predict the outcome added more benefit than a treat-all-patients or treat-none scheme if the threshold probability is between 0.2 and 0.6 in both cohorts.

## Discussion

In this retrospective study, we identified a close correlation between the relative signal intensity attenuation index (rSIAI) from arterial to portal phase (rSI_ap) and disease control in HCC patients who received synchronous combined systemic treatment with anti-angiogenesis and ICI. The results also showed that rSI_ap, in combination with the CD4+ lymphocyte subpopulation in peripheral blood, performed better in predicting disease control compared to rSI_ap or CD4+ alone. Furthermore, we constructed a novel nomogram based on rSI_ap and CD4+. Calibration and decision curve analyses confirmed the clinical relevance and value of the nomogram. We believe that the nomogram has the potential to assist clinicians in making clinical decisions.

Qualitative MRI characteristics have been extensively utilized in differentiating liver tumors from tumor-like lesions (17–19). Recent studies have shown that quantitative parameters derived from CEMR are useful in predicting clinical outcomes for patients with liver tumors. Gu et al. demonstrated that the relative tumor enhancement and the standard deviation ratio can predict the pathological response to systemic therapy in patients with colorectal liver metastases (20). In a study by Ahn et al., the authors assessed the predictive value of preoperative gadoxetic acid-enhanced MR imaging. The results indicated that peritumoral hypointensity during the hepatobiliary phase could predict early recurrence in patients with HCC (21). In these studies, quantitative parameters were calculated based on the signal intensity observed during the hepatobiliary phase of Gd-EOB-DTPA-enhanced MRI. Moreover, the parameters used were static, meaning they were measured at a fixed phase, which fails to capture the dynamic changes in signal intensity over time.

In this study, the inter-observer reliability for measuring signal intensity was found to be excellent, with ICCs exceeding 0.9. This indicates a high level of repeatability for the measurement method used. We calculated the relative signal intensity (rSI) and the rSI attenuation index (rSIAI) over time to ensure that our results could be applied to various MRI brands. The dynamic nature of signal intensity more accurately reflects blood flow and venous drainage in tumors compared to static parameters. The relative signal intensity during the arterial phase (rSI_ap) provides insight into the velocity of venous drainage and portal vein perfusion within the tumor microvasculature. A lower rSI_ap, which is associated with slower venous drainage or increased perfusion from the portal vein within the tumor microenvironment (TME), may provide more opportunities for cytotoxic lymphocytes to infiltrate the TME and enhance anti-tumor efficacy. CD4+ T cells can help promote the efficacy of cytotoxic T cells and have been proven to be associated with better outcomes in HCC patients. Our results indicate that higher CD4+ T cells correlate with better outcomes, which is consistent with those reported. Previous studies showed that higher counts of peripheral blood CD3+ and CD8+ T cells at baseline were linked to improved outcomes in HCC patients receiving immunotherapy (22, 23). The role of the CD4+/CD8+ ratio as a predictor in cancers is disputed by the existing evidence (24, 25). In our study, there were no statistically significant differences in CD3+, CD8+ T cells, and the CD4+/CD8+ ratio between the groups, which may be attributed to the small sample size of the cohort. Additionally, the clinical significance of T-cell subsets may be influenced by the body’s immune function and underlying conditions, such as viral infections and autoimmune diseases (26, 27).

This study has several limitations. First, it is a single-center, retrospective study with a small sample size, which may introduce unintentional biases and affect the generalizability of the results. Second, there was no predictive performance or external validation to confirm the findings. Third, the MRI images were taken in a transverse view, limiting their applicability for multiplanar reconstruction (MPR). As a result, the ROIs in images from different enhancement phases may not align precisely. Further investigation is needed to determine whether this slight misalignment impacts the predictive accuracy of the nomogram.

## Conclusion

In conclusion, this is the first study to construct and assess a novel nomogram based on quantitative CEMR parameters in combination with peripheral CD4+ lymphocyte subpopulations to identify HCC patients who might benefit from combined systemic treatment of anti-angiogenesis and ICI. Our findings provide useful information for improved and individualized treatment options in patients with HCC, which is valuable for patient management.

## Data Availability

The original contributions presented in the study are included in the article/**Supplementary Material**. Further inquiries can be directed to the corresponding author.
