# Supplementary material for: A nomogram based on quantitative MR signal intensity predicts early response to combined systemic treatment in patients with hepatocellular carcinoma
Source: Front Oncol. 2025 Mar 13;15:1527108. doi: 10.3389/fonc.2025.1527108 (PMC11959652; doi:10.3389/fonc.2025.1527108)
Supplement: Supplementary file 1 [file DataSheet1.docx]

***Supplementary Material***

1. **Supplementary Data**

## MR data acquisition

All imaging data were acquired using Siemens magnetic resonance imaging (MRI) scanners operating at field strengths of 1.5T or 3T. Multiphase dynamic contrast-enhanced (DCE) imaging was performed using T1-weighted Volumetric Interpolated Breath-hold Examination (VIBE) sequences combined with Dual-Echo Imaging with Chemical Shift Effect (DXION) technology. The imaging parameters were standardized as follows: slice thickness = 3.0 mm; slice gap = 0 mm; field of view (FOV) = 400 mm × 333 mm; voxel size = 1.6 mm × 1.79 mm; reconstructed voxel size = 0.7 mm × 0.7 mm × 1.5 mm; and acquisition time per phase = 12 seconds.

The echo time (TE) and repetition time (TR) were optimized according to the magnetic field strength: for 3T scanners, TE = 1.29/2.52 ms and TR = 3.97 ms; for 1.5T scanners, TE = 2.39/4.78 ms and TR = 6.91 ms. These variations in TE and TR were attributed to the intrinsic physical constraints associated with the respective field strengths.

For contrast-enhanced imaging, a high-pressure injector was utilized to administer gadolinium-based contrast agent at a dose of 0.2 mmol/kg body weight, delivered at an injection rate of 3–4 ml/s, followed by a 20 ml saline flush administered at the same rate.

1. **Supplementary Figures and Tables**

## Supplementary Figures


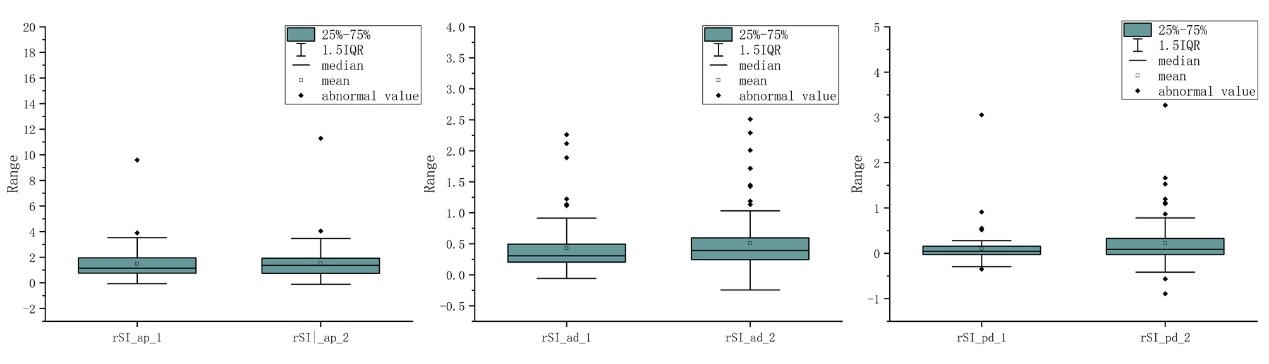


**Supplementary Fig 1.** The details of the rSIAIs (rSI_ap, rSI_ad, and rSI_pd).


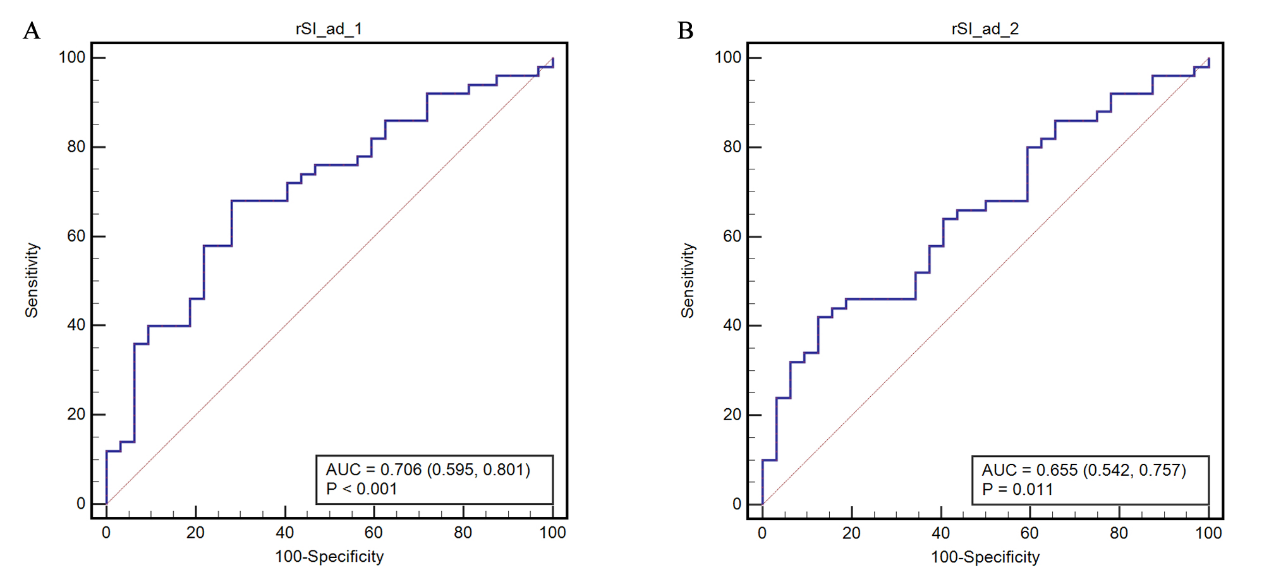


**Supplementary Fig 2.** ROC curves for predicting disease control based on the COVs of rSI-ad (A and B) of the two observers.

## Supplementary Table 1. Intra-class Correlation Coefficient analysis

|  | **Variables** | **ICC** | **95% CI** | |
| --- | --- | --- | --- | --- |
| **Measured** | nSI_Liver | 0.998 | 0.9969 | 0.9987 |
|  | aSI | 0.9429 | 0.9136 | 0.9624 |
|  | pSI | 0.9673 | 0.9507 | 0.9784 |
|  | dSI | 0.9433 | 0.9172 | 0.9614 |
| **Calculated** | raSI | 0.9272 | 0.8897 | 0.9523 |
|  | rpSI | 0.9409 | 0.9101 | 0.9614 |
|  | rdSI | 0.7973 | 0.7121 | 0.8593 |
|  | rSI_ap | 0.9407 | 0.9107 | 0.9609 |
|  | rSI_pd | 0.6363 | 0.51 | 0.7358 |
|  | rSI_ad | 0.8907 | 0.8402 | 0.9258 |
